# Methanol, Paracetamol Toxicities and Acute Blindness

**DOI:** 10.7759/cureus.8179

**Published:** 2020-05-18

**Authors:** Aldanah Althwanay, Manar M Alharthi, Mohammad Aljumaan, Yousef Almubarak, Abdullah Alamri

**Affiliations:** 1 Internal Medicine, Imam Abdulrahman Bin Faisal University, Khobar, SAU; 2 Internal Medicine, King Fahad Specialist Hospital, Dammam, SAU; 3 Emergency Medicine, Imam Abdulrahman Bin Faisal University, Khobar, SAU; 4 Neurology, Imam Abdulrahman Bin Faisal University, Khobar, SAU

**Keywords:** methanol toxicity, paracetamol toxicity, blindness, coma, dialysis

## Abstract

Methanol toxicity remains a common problem in developing countries including Saudi Arabia. However, it is much less available than other toxins; thus, clinical suspicion and correlation play pivotal roles in diagnosis. On the other hand, paracetamol is widely available and overdose/toxicity is highly suspected especially in young females. Diagnosis of methanol toxicity can be difficult in cases where history is not readily available and it requires a high degree of suspicion, especially when ingestion of another substance is revealed first as should not preclude the possibility of co-ingestion.

We report a case of a medically free 26 year old woman who was brought to the Emergency Department (ED) of our hospital with a history of ingestion of a massive amount of paracetamol tablets and a concurrent abdominal pain with vomiting. After arrival, she became unresponsive with Glasgow Coma score (GCS) of 8/15 and was mechanically ventilated. Initial laboratory investigations identify the paracetamol level of 1200 ug/ml and a significantly high anion gap metabolic acidosis. Owing to the suspicious circumstances and the depth of acidosis, a co-ingestion of methanol and ethylene glycol was suspected. Upon further evaluation, osmolal gap was found to be significantly high as well. Since neither volatile compound screening nor Fomepizole were available in our hospital, an urgent N-acetylcysteine and sodium bicarbonate (NaHCO_3_) were started alongside hemodialysis. Subsequent brain computed tomography (CT) and magnetic reasoning imaging (MRI) revealed changes in putamen and basal ganglia most consistent with methanol toxicity. She was successively extubated on day four of hospitalization with residual visual deficits that had resolved eventually after several follow-ups.

## Introduction

Methanol is a clear and colorless form of toxic alcohol. In the developing countries including Saudi Arabia, it can be found as a solvent in some brands of perfume and cologne, handmade liquor and smuggled alcohol. Ingestion of methanol may be accidental or as a suicidal attempt [1]. Methanol toxicity remains a common problem, however, it is much less available than other toxins; thus, clinical suspicion and correlation play pivotal roles in diagnosis. On the other hand, paracetamol is widely available and overdose/toxicity is highly suspected especially in young females. The presentation of methanol toxicity can be vary from mild to life-threatening symptoms similar to that of paracetamol toxicity; hence symptoms may overlap and lead to a premature diagnosis of ingestion of a single specific substance without ruling out the possibility of co-ingestion of a single specific substance and the consequences of their interactions. The literature does not discuss the possible consequences and interactions of methanol and paracetamol co-ingestion. However, an article published in 2008 revealed that the co-ingestion of paracetamol and ethanol, which is another form of alcohol, actually limits the effect of toxicity of both substances together [2]. Exposure to methanol can cause a wide range of neurological manifestations, most commonly confusion, coma and vision loss and less frequently tremor as a manifestation of putaminal hemorrhage/necrosis. Reports have indicated that death from methanol toxicity had ranged from 8-36% and permanent loss of vision has been observed in 20-40% of patients who survive the acute injury [3-5]. Paracetamol overdose can cause non-specific symptoms within the first 24 hours, including fatigue, abdominal pain, or nausea. After several days, patients may complain of jaundice, blood clotting problems, and confusion that occurs as a result of liver failure. Additional complications may include kidney failure, pancreatitis, low blood sugar, and lactic acidosis [6].

## Case presentation

**Figure 1:**
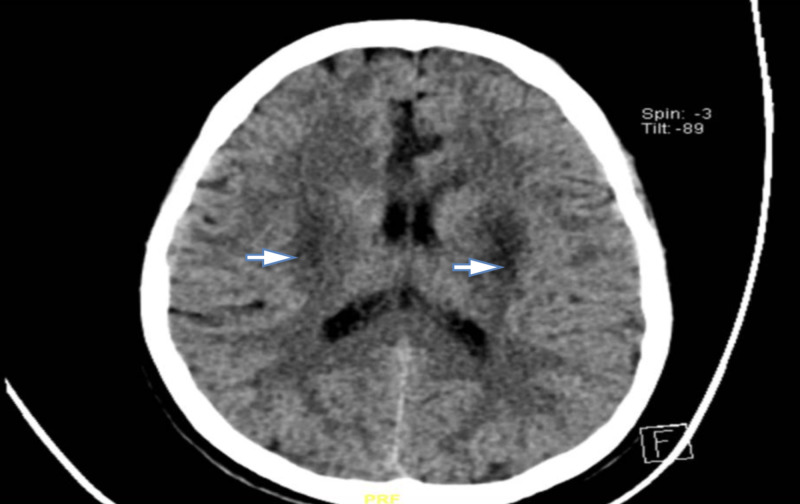
Non-enhanced CT scan of the brain showing bilateral basal ganglia hypodensities

**Figure 2:**
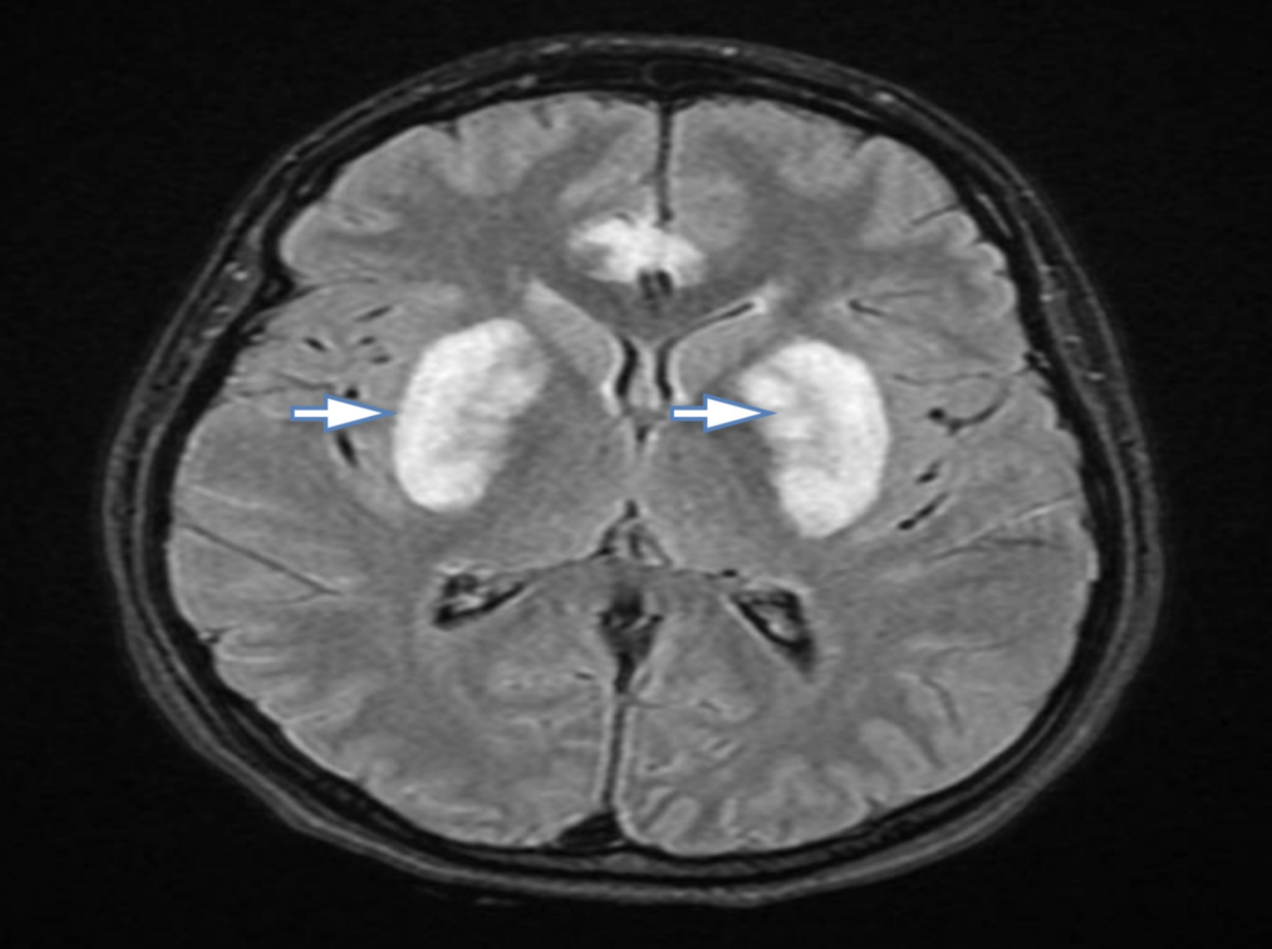
Cranial MRI showing multiple hyperintensities including basal ganglia

A 26 year old healthy female presented to our ED with a one-day history of a massive amount of paracetamol tablets ingestion and a concurrent abdominal pain with vomiting. Her condition promptly deteriorated and she became confused and unresponsive. Upon presentation, her blood pressure was 93/60 mmHg, pulse rate was 130 bpm, respiratory rate was 30 Bpm, SaO2 was 87% on room-air, random blood sugar was 140 mg/dl and GCS of 8/15. On examination, she had fixed bilaterally dilated pupils (6 mm). A baseline electrocardiogram (ECG) was taken and showed supraventricular tachycardia alongside hemodynamic instability for which she received two synchronized cardioversion shocks. She was immediately resuscitated with intravenous fluid and was intubated. Initial laboratory results including liver function test were within acceptable ranges except for pH (6.68), anion gap (31mmol/ L), delta anion gap (0.9 mmol/L), PCO2 (24.7mmHg), HCO_3_: (3.9mmol/L), creatinine level (1.5mg/dl), potassium (6.6mmol/L), lactic acid (6.4 mmol/L), urine ketones (15 mg/dl). Urine toxicology for was positive for acétaminophèn (1200 ug/ml after 12 hours of ingestion) and salicylate (less than 0.2 ug/ml), but was negative for opiates. The presence of high anion gap metabolic acidosis was initially thought to be secondary to ketoacidosis and hyperlactatemia. However, the degree of lactic acidosis and ketonuria was not high enough to explain the extent of the acidosis and on further evaluation, osmolal gap was calculated to be 34.1 mmol/L. Since the osmolal gap was high, toxic alcohol ingestion (most probably methanol) was the working diagnosis owing to the dubious circumstances and clinical presentation. Methanol level was not obtained as our hospital lacks a volatile compound screening test. A head CT followed by MRI were obtained after initiating treatment and revealed putaminal and basal ganglia necrosis consistent with methanol toxicity (Figure 1, 2).

As the patient had severe metabolic acidosis with pH < 7.1, she was given NaHCO3 infused with 5% dextrose and was put on urgent hemodialysis to clear the methanol as Fomepizole is not available in our hospital regularly. In addition to that, she received N- acetylcysteine for paracetamol toxicity and was admitted to the Intensive Care Unit (ICU). On the second day of her admission and during the time of sedation vacation, visual threat examination revealed vision loss and the ophthalmologist confirmed the diagnosis of toxic optic neuropathy. On the fourth day, successful extubation was achieved with residual visual deficits. Her blood pH, anion gap and osmolal gap levels had normalized eventually. Follow up appointments with neurology, psychiatry and ophthalmology clinics were recommended. After several follow-ups, she regained her full vision.

## Discussion

Methanol toxicity remains a common problem in developing countries including Saudi Arabia. Methanol gets oxidized by alcohol dehydrogenase to formaldehyde, which is oxidized further to formic acid by formaldehyde dehydrogenase. Formic acid accumulates are responsible for metabolic acidosis in methanol toxicity cases and there is a direct correlation between formic acid concentration and incidence of morbidity and mortality. Formic acid inhibits cytochrome oxidase and is the cause of ocular toxicity [7]. The presentation of methanol toxicity can vary from mild symptoms to life-threatening. At first, symptoms of methanol toxicity could be similar to alcohol toxicity. For instance, the patient may present with ataxia, disinhibition, nausea, vomiting, and epigastric abdominal pain. Drowsiness could rapidly progress to seizure and coma in later stages due to metabolic acidosis indirectly or by direct damage to the brain parenchyma, eventually requiring intubation [8]. Likewise, methanol toxicity may present similar to paracetamol toxicity, where patients present with abdominal pain, nausea, vomiting and in severe cases metabolic acidosis secondary to lactic acidosis [6]. Our patient, for instance, presented with abdominal pain and vomiting alongside hemodynamic instability. Her condition rapidly deteriorated where she became unresponsive and developed supraventricular tachycardia for which she was rapidly resuscitated with two synchronized cardioversion shocks in addition to intravenous fluid and endotracheal intubation.

One of the most common manifestations of methanol toxicity is ocular findings which include optic disc hyperemia, compromised pupillary response with little or no retinal damage [8]. Our patient had early presentation of fixed bilateral dilation of pupils. An ophthalmology examination had revealed bilateral vision loss. Serum methanol levels and arterial blood gas are crucial for diagnosis. Moreover, the anion gap and osmolal gap calculations are essential to differentiate between methanol toxicity and other causes of high anion gap metabolic acidosis such as ketoacidosis and lactic acidosis [9,10].

Both the high anion gap and osmolal gap suggested toxic alcohol ingestion such as methanol and ethylene glycol [11]. Diagnosis can be difficult in cases where history is not readily available and requires a high degree of suspicion, especially when ingestion of another substance is revealed first as it should not preclude the possibility of co-ingestion. Our patient had presented with paracetamol ingestion and co-ingestion of methanol was highly suspected, especially in such patients with high-risk behavior alongside the significantly high anion gap and osmolal gap which could be explained by the extent of neither ketoacidosis nor lactic acidosis.

Since none of the published literature discussed or reported the co-ingestion of paracetamol and methanol together, it is unknown if there was a possible interaction between them and whether it would affect the extent of toxicity of both substances negatively or positively.

Brain imaging may help by showing the characteristic findings of bilateral putaminal necrosis/ hemorrhage [8]. Nevertheless, these findings are not specific and can be seen with other diseases, such as Wilson's disease and stroke [12]. Prompt treatment with Fomepezole or hemodialysis is crucial as the degree and irreversibility of damage caused by formic acid is time-sensitive and the increased acidity facilitates the diffusion of formic acid into cells [8]. Thus, our patient received NaHCO_3_ to decrease the acidity as pH cutoff was ≤ 7.1 and underwent hemodialysis to eliminate both methanol and co-ingested paracetamol alongside N-acetylcysteine.

## Conclusions

Diagnosis of methanol toxicity can be difficult in cases where history is not readily available and requires a high degree of suspicion, especially when another substance ingestion is revealed first as it should not preclude the possibility of co-ingestion. Prompt treatment with Fomepezole or hemodialysis is crucial as the degree and irreversibility of damage caused by formic acid is time-sensitive.
